# Leiomyosarcoma of the stomach with metastasis to the liver: a case report with review of the literature

**DOI:** 10.4155/fsoa-2017-0100

**Published:** 2017-11-20

**Authors:** Varshil Mehta, Monali Rajawat, Sameer Rastogi, Ravi H Phulware, Roman Mezencev

**Affiliations:** 1 Department of Internal Medicine, MGM Medical College, Navi Mumbai, India; 2 Department of Cardiology, Mount Sinai Hospital, New York, NY 10029, USA; 3 Department of Internal Medicine, R.N.T. Medical College, Udaipur, India; 4 Department of Oncology, All India Institute of Medical Science, New Delhi, India; 5 Department of Pathology, All India Institute of Medical Science, New Delhi, India; 6 School of Biological Sciences, Georgia Institute of Technology, Atlanta, GA 30332, USA

**Keywords:** docetaxel, doxorubicin, gastrointestinal stromal tumors, gemcitabine, GIST, leiomyosarcoma, pazopanib, soft tissue sarcoma

## Abstract

Leiomyosarcoma of the stomach is a very rare malignancy that was not distinguished from the more frequent gastrointestinal stromal tumors until early 2000s. Here we report on a case of a metastatic disease that developed in a 47-year-old man 2 years after he was diagnosed with the primary tumor and treated with curative surgical excision and adjuvant doxorubicin. The primary and metastatic lesions were positive for smooth muscle markers α-smooth muscle actin and h-caldesmon and negative for CD117, DOG-1 and S100 by immunohistochemistry. Metastatic disease progressed on additional monotherapy with doxorubicin and docetaxel–gemcitabine combination, and stable disease was achieved upon treatment with pazopanib. Patient is surviving 35 months since diagnosis of the primary tumor and 11 months since diagnosis of metastatic disease.

## Lay abstract

Sarcomas are cancers of connective tissues that are much less common than epithelial cancers. Leiomyosarcoma is a type of sarcoma derived from smooth muscle cells, which can grow almost anywhere in the body, but it is extremely rare in some sites. Because it is so rare, it is important to discuss leiomyosarcoma cases within medical and scientific community. These discussions improve diagnosis and treatment and foster scientific research of these rare cancers. In this report we discuss diagnosis and treatment of a patient with leiomyosarcoma of the stomach, whose disease progressed 2 years after he was disease-free following initial treatment.

## Graphical abstract

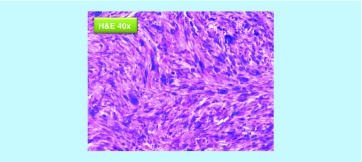

Sarcomas are malignant mesenchymal neoplasms that represent less than 1% of all solid malignant tumors in adults [1]. Considering numerous anatomical sites, more than 70 histological types and increasing number of molecular subtypes, sarcomas are a remarkably heterogeneous group of diseases [2] that pose disproportionately high diagnostic and therapeutic challenges in clinical practice. Leiomyosarcomas (LMS) are composed of cells that display smooth muscle features and account for about 11% of all soft tissue sarcomas (STS) [3]. They are usually diagnosed in middle-aged or older individuals and arise in a number of anatomical sites that include uterus, retroperitoneum, skin, peripheral soft tissue (noncutaneous, nonretroperitoneal) and bone, but also some extremely rare primary locations, such as the thyroid gland, gallbladder, liver, bronchus and pancreas [4].

In the gastrointestinal tract (GIT), LMS are rare and far less prevalent malignant mesenchymal tumors than gastrointestinal stromal tumors (GIST), which originate from the interstitial cell of Cajal, but display similar gross and microscopic morphology to LMS, which originate from smooth muscle cells within the muscularis mucosa or muscularis propria. Due to this morphological similarity, GIST have been previously diagnosed as GIT LMS, before they were recognized as a distinct entity with molecular profiles and clinical behaviors that differ from true smooth muscle-derived tumors and require different therapeutic approaches [5]. LMS are extremely rare in stomach and most cases or case series reported in the ‘pre-KIT era’ as LMS of the stomach actually represented GIST of the stomach [6]. As a result, understanding the biology and clinical behavior of LMS of the stomach is very limited and presentation of new cases is highly desirable.

## Case report

**Figure 1.**
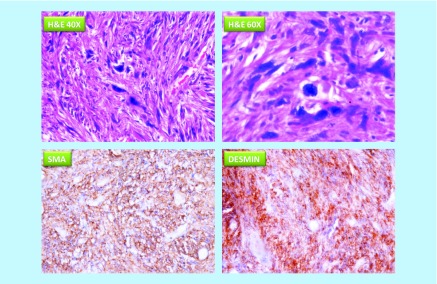
**Micrographs of primary tumor stained with haematoxylin–eosin (H&E 40X and H&E 60X), and immunohistochemistry for α-smooth muscle actin and desmin (Desmin).** The images demonstrate the presence of spindle-shaped tumor cells with eosinophilic cytoplasm and hyperchromatic pleomorphic nuclei with numerous mitotic figures. Cells showed cytoplasmic positivity for α-SMA and desmin. H&E: Haematoxylin–eosin; SMA: Smooth muscle actin.

A 47-year-old man with unremarkable past medical history, no use of tobacco, alcohol, immunosuppressants or corticosteroids, and no known exposure to industrial chemicals or ionizing radiation, presented with pain in the left hypogastric region that was present for the last 10 days with severity 7 out of 10 on the pain assessment scale. Ultrasound examination identified well defined hypoechoic lesion with dimensions 118 mm × 107 mm × 77 mm. 2 months later, CT scan displayed exophytic solid mass arising from the greater curvature of the body of the stomach with heterogeneous contrast enhancement and dimensions 83 mm × 114 mm × 111 mm. The lesion was supplied by multiple jejuna branches of superior mesenteric arteries. The patient underwent laparotomy with total excision of the greater curvature of the stomach. The macroscopic examination found brownish multinodular mass with dimensions of 130 mm × 130 mm × 100 mm. The microscopic examination with haematoxylin–eosin staining found spindle shaped tumor cells with eosinophilic cytoplasm and hyperchromatic pleomorphic nuclei with numerous mitotic figures. Immunohistochemistry was positive for α-smooth muscle actin (α-SMA), desmin (Figure 1), and h-caldesmon, but negative for CD117, DOG-1 and CD34. Pathologic staging was pT2bN0M0 (stage III). The patient received adjuvant chemotherapy with six cycles of doxorubicin in monotherapy (90 mg/cycle) and subsequently remained free of clinically detectable tumors for the two following years.

**Figure 2.**
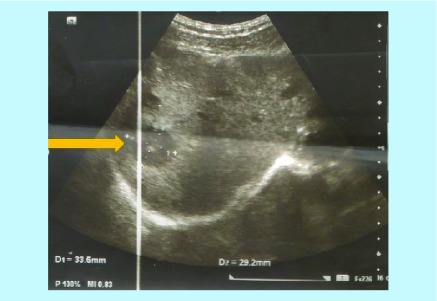
**Ultrasonography showing the largest lesion in the liver 24 months postsurgery.**

**Figure 3.**
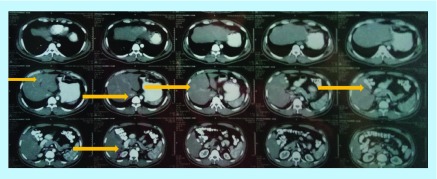
**CT scan performed 24 months postsurgery displays multiple hypodense lesions in liver.**

**Figure 4.**
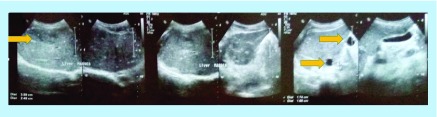
**Ultrasonography showing multiple hepatic lesions after two cycles of doxorubicin monotherapy.**

**Figure 5.**
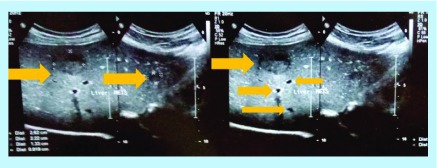
**Ultrasonography showing the largest lesion in the liver 30 months after surgery for primary leiomyosarcomas.**

**Figure 6.**
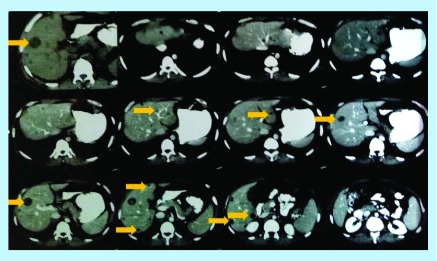
**CT scan performed 30 months after surgery for primary leiomyosarcomas showing multiple hypodense lesions in the liver.**

After 2 years, a follow-up imaging by ultrasound (Figure 2) and CT scan (Figure 3) displayed multiple hypoechoic lesions in both liver lobes with largest lesion size of 33.5 mm × 29.2 mm. Biopsy of the hepatic lesions confirmed the presence of spindle-shaped tumor cells with eosinophilic cytoplasm and nuclear pleomorphism. Immunohistochemistry (IHC) was positive for α-SMA and h-caldesmon, but negative for desmin, S100, c-kit and DOG-1, which confirmed LMS as the origin of these metastatic lesions. The patient received two cycles of doxorubicin in monotherapy, but showed disease progression after 2 months on chemotherapy with increasing number of small hepatic lesions (<1 cm diameter) and with largest lesion measuring 35 mm × 24 mm (Figure 4). Subsequently, the chemotherapy was changed to six cycles of gemcitabine–docetaxel combination, starting with gemcitabine (1400 mg) on day 1 followed by a combination of gemcitabine (1200 mg) with docetaxel (100 mg) on day 8. In the course of this treatment, the largest lesion regressed (by ultrasonography; USG [Figure 5]) to 26 mm × 22 mm, which represents only about 22% decrease in diameter, compared with the size of this lesion before the initiation of gemcitabine–docetaxel chemotherapy; however, many new metastatic lesions occurred in the liver (Figures 5 & 6). As a result, this regimen did not impede the disease progression.

**Figure 7.**
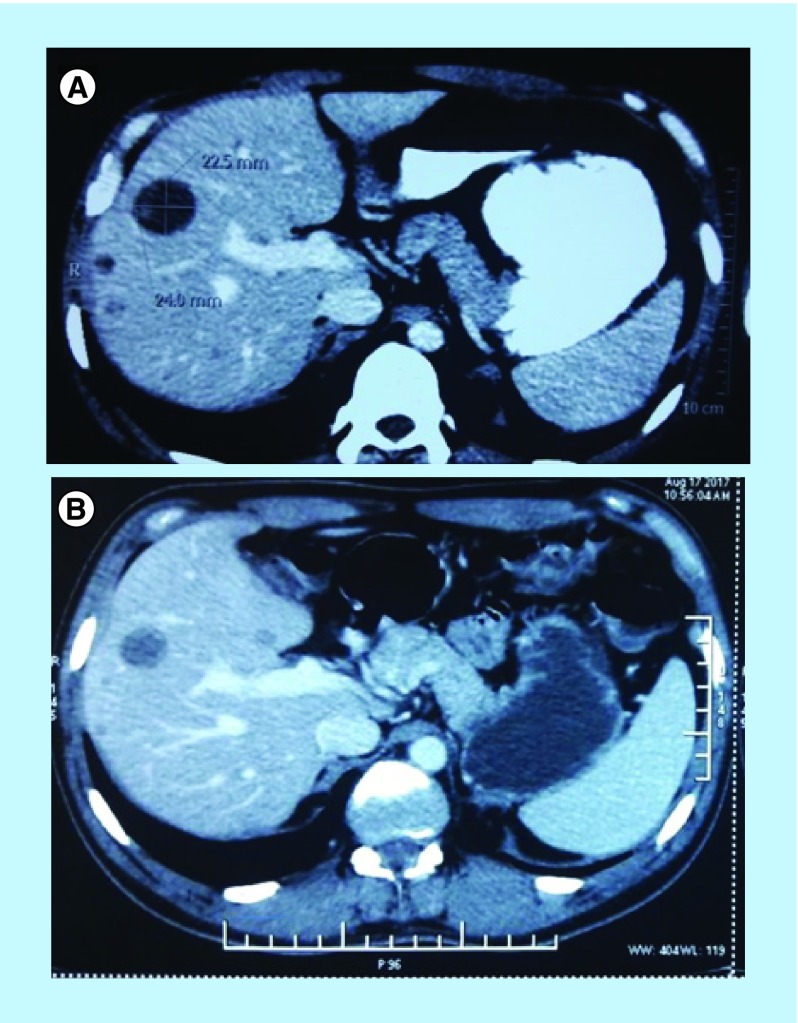
**CT imaging of abdomen.** **(A)** Prior initiation of pazopanib treatment; and **(B)** 12 weeks after the initiation of pazopanib treatment.

For this reason, the patient was prescribed pazopanib, a selective multikinase inhibitor, which was approved in 2012 by the US FDA for the treatment of patients with advanced soft tissue sarcomas who have received prior chemotherapy. The initial dose was set to 400 mg/day for 1 month and subsequently increased to 600 mg/day, but it had to be decreased to 400 mg/day again due to palmar-plantar erythrodysesthesia that developed presumably as a side effect of this therapy. 12 weeks after the initiation of treatment with pazopanib, the CT scan of the whole abdomen was performed with and without intravenous nonionic contrast, and images were acquired in arterial, hepatic and delayed phase. Numerous hypodense lesions were identified in all segments of liver with lower enhancement in all phases in comparison with the liver parenchyma. CT scan before initiation of treatment displayed largest lesion with dimension of 24.0 mm × 22.5 mm (Figure 7A), while the largest lesions measured 20 mm × 18 mm (Figure 7B) post 12 weeks of initiation of the therapy. No new metastatic lesions developed in the liver after pazopanib treatment was initiated. The limited reduction of diameter of the major metastatic lesion approximately 17% implies stable disease. The patient remains alive after 35 months since diagnosis of primary LMS and 11 months since detection of liver metastases and continues treatment with oral pazopanib at 400 mg/day.

## Discussion with review of literature

LMS is one of the most common primary retroperitoneal malignant mesenchymal tumor in adults [7], but at the same time it is a rare disease associated with considerable diagnostic and therapeutic challenges. Its etiology is not yet clear, but ionizing radiation, chemical exposures, Epstein–Barr virus and immunosuppression have been suggested to play role in the development of LMS and other STS [1,8,9].

LMS can occur in many different anatomical sites and the clinical outcome seems to depend considerably on the site of origin, in spite of high histological similarity among LMS from different anatomical locations. For instance, patients with uterine LMS tend to present with larger tumors and more frequently with metastatic disease at the time of diagnosis, when compared with patients with nonuterine LMS, which accounts for their less favorable outcome [10].

Likewise, the primary site of LMS appears to have considerable influence on the response to chemotherapy. According to the Eastern Cooperative Oncology Group and Southwest Oncology Group, response rate to chemotherapy was 20–25% in uterine leiomyosarcomas, but about twice-lower response rate was observed in leiomyosarcomas of the GIT. Even though many of these LMS would now be classified as GIST, genuine LMS of the GIT appear to have lower response to chemotherapy than LMS arising from other sites [11].

The existence of biological differences between LMS from distinct anatomical sites is subject of ongoing discussions. Undeniably, leiomyosarcomas display varying degrees of smooth muscle differentiation and complex genetic abnormalities. If true and recurrent biological differences are demonstrated, they may allow future development of personalized therapeutic approaches. A recent study demonstrated existence of three leiomyosarcoma molecular subtypes with different gene expression profiles, including expression of genes for which targeted therapies are being developed [12]. Nevertheless, surgical resection provides the only curative option for LMS presently, and conventional chemotherapy and radiotherapy display only limited efficacy. Systematic therapy in patients with metastatic LMS is not selected based on the site of LMS origin or its underlying biology.

GIST have been previously diagnosed as LMS until early 2000s, when GIST became recognized as a distinct entity. Since then, LMS of the GIT can be distinguished from GIST, which is a far more prevalent mesenchymal malignancy in the GIT [5]. This differentiation is based on the expression of KIT receptor (CD117), which is expressed in tumor cells in the overwhelming majority of GIST cases, as well as DOG-1 that is expressed in some CD117-negative GIST cases. Another immunohistochemical marker, which is frequently used for diagnosis of GIST, is CD34, even though it is neither sensitive nor specific to distinguish GIST from other mesenchymal neoplasms [11]. In contrast, LMS are generally negative for KIT or DOG-1 and frequently positive for α-SMA, desmin, calponin, h-caldesmon or smoothelin. Differentiation between GIST and gastrointestinal LMS is of critical importance, considering that GIST usually responds to KIT-directed therapy with imatinib or sunitinib, while expression of drug resistance proteins MDR1 and MRP1 in GIST contributes to their intrinsic resistance to many conventional anticancer drugs [11,13].

Our patient met IHC diagnostic criteria for LMS with expression of α-SMA, h-caldesmon and desmin in primary tumor cells and negative IHC for CD117, DOG-1, CD34 and S100. IHC was negative for desmin in the liver metastatic lesion, which demonstrates variability in expression of markers of smooth muscle differentiation in LMS. This difference may be biologically relevant, but it was not significant for the diagnosis of LMS in our case, considering positive expression of other markers of smooth muscle differentiation.

Imaging modalities assist in diagnosis of LMS; however, they cannot provide reliable definitive diagnosis. Diagnostic USG is a readily available and inexpensive method that is generally used for the early evaluation of soft tissue masses, providing initial evaluation of the size, location and consistency of soft tissue lesions. In addition, it is able to differentiate between cystic and solid lesions and USG may detect local tumor recurrences through the presence of hypoechoic lesions. On the other hand, USG is not useful for measurement of tumor sizes to assess tumor burden and the response to therapy, which is emphasized by the Response Evaluation Criteria In Solid Tumors (RECIST) guideline [14]. Nevertheless, more advanced USG techniques, such as color doppler and 3D-USG may provide valuable addition to the conventional USG and improve the acceptance of USG in clinical practice [15].

CT scan is the primary imaging modality for the assessment and evaluation of LMS and the presence and extent of metastatic disease [16]. Most LMS grow silently and they are often diagnosed when their size becomes large with extensive necrotic or cystic changes [16,17]. MRI can also be utilized to define exact location of the primary tumor and its extent in relation to blood vessels, nerves and other structures [4,7,16]. Modern imaging techniques, such as diffusion weighted MRI and ^18^FDG-PET, have greater sensitivity to distinguish malignant and benign lesions, which can be helpful in the diagnosis of leiomyosarcomas [4] that occur far less often in GIT than benign leiomyomas [18].

LMS have predilection for hematogenous spread. In approximately 40% of patients, distant metastases are present at the time of initial diagnosis and most patients eventually develop metastatic disease [8]. Liver and lungs are the most common sites of metastasis [16,17]; however, LMS is also known to metastasize to the stomach, pancreas, small bowel, cardiac chambers, skin, submandibular salivary gland, scalp, skeletal muscles and subcutaneous tissue [8,19–28]. Pancreas is a rare site for metastasis of LMS and only less than 20 cases of pancreatic metastasis from LMS have been reported so far [19–21].

Hepatic resection for liver metastases in combination with chemotherapy has been established as a standard treatment of LMS metastasizing to the liver. However, due to the presence of multiple lesions, our patient was not a suitable candidate for hepatic resection. Furthermore, probability of disease recurrence is high even after the resection of metastatic lesions. DeMatteo *et al*. concluded that even after complete hepatic resection of sarcoma metastases, the rate of recurrence was as high as 84% [29]. For this reason, we decided to pursue chemotherapy for treatment of metastatic LMS in our patient.

At the present time, European Society for Medical Oncology (ESMO) and the National Comprehensive Cancer Network (NCCN) treatment guidelines recommend anthracycline-based chemotherapy, primarily anthracyclines alone or in combination with ifosfamide for soft tissue sarcomas, although standardized chemotherapy for LMS specifically has not yet been established. Moreover, gemcitabine and docetaxel combination is also used [30–32]; however, response rates of LMS are usually just 10–25% [33]. In a nationwide study conducted by Choi *et al*., the response was observed in 26.3% of the LMS patients, with median overall survival (OS) and progression-free survival (PFS) of 10.3 months (95% CI: 8.4–12.2) and 3.3 months (95% CI: 2.8–4.7), respectively [34]. Nevertheless, in the presented case, the combination gemcitabine–docetaxel did not decrease tumor burden and did not result in stabile disease either.

More recently, a marine alkaloid trabectedin, which interferes with transcription and DNA repair, has also proven to be an effective salvage therapy for the patients with advanced STS previously treated with doxorubicin and/or ifosfamide [32]. In a small Phase II study of 36 patients with metastatic or recurrent soft tissue sarcoma, trabectidin induced 17% response rate with median PFS of 1.6 months, median OS of 15.8 months and 1 year overall survival rate of 72% [33].

As per the most recent studies, pazopanib, which inhibits multiple tyrosine kinases, including VEGFR-1, -2, and -3, PDGFR-α and -β, FGFR-1 and -3, c-kit, IL-2 receptor-inducible T-cell kinase, leukocyte-specific protein tyrosine kinase, and transmembrane glycoprotein receptor tyrosine kinase (c-fms) have shown promising results in treating LMS [35]. A Phase III clinical trial of the European Organization for Cancer Research ‘PALETTE’ compared placebo and pazopanib in the treatment of metastatic soft tissue sarcomas. The trial enrolled both nonuterine and uterine LMS patients in the double-blind randomized placebo-controlled multicenter study. The patients were randomized to placebo or pazopanib 800 mg once-daily arms. Median PFS was 4.6 months (95% CI: 3.7–4.8) for pazopanib versus 1.6 months (95% CI: 0.9–1.8) for placebo (p < 0.0001), and the median OS was 12.5 months (95% CI: 10.6–14.8) with pazopanib versus 10.7 months (95% CI: 8.7–12.8) with placebo (p = 0.25). This trial established pazopanib as a new treatment option for patients with nonadipocytic metastatic STS who have previously been treated with chemotherapy [36]. The overview of other chemotherapeutic regimens previously reported for STS is provided in Table 1.

**Table 1.** **Comparison among different chemotherapy regimens.**

**Sr. No.**	**Study**	**Year**	**Country of the patient**	**Drug**	**Progression-free survival**	**Overall survival**	**Response rate**	**Ref.**
1	Nakamura *et al*.	2016	Japan	Pazopanib	18.6 weeks	20.1 months	44% (PR or SD)	[37]

2	Oosten *et al*.	2009	Netherland	Doxorubicin	3.7 months	9.7 months	18% (CR + PR)	[38]

3	Harter *et al*.	2014	Essen	Gemcitabine	3 months	11.5 months	–	[39]

				Gemcitabine + Docetaxel	6.2 months	17.9 months		

4	Choi *et al*.	2017	Korea	Gemcitabine + Docetaxel	3.3 months	10.3 months	15.1%	[34]

5	Tap *et al*.	2016	USA	Olaritumab + Doxorubicin	6.6 months	26.5 months	18.2%	[40]

				Doxorubicin	4.1 months	14.7 months	11.9%	

6	Le Cesne *et al*.	2005	France	Ecteinascidin (ET-743)	6 months	9.2 months	56% (PR + No change)	[33]

7	Demetri *et al*.	2016	–	Trabectedin	4.2 months	13.5 months	34% (PFS: 6 months)	[41]

				Dacarbazine	1.5 months	10 months	14% (PFS: 6 months)	

8	Demetri *et al*.	2013	–	Ridaforolimus	17.7 weeks	90.6 weeks	PR and SD: 4 Months	[42]

9	Demetri *et al*.	2009	–	Trabectidin	3.3 months	13.9 months	<33%	[43]

CR: Complete response; OS: Overall survival; PFS: Progression-free survival; PR: Partial response; SD: Stable disease.

New regimens for the treatment of LMS are under development. For instance, a Phase II study showed a significant difference in overall survival between patients treated with doxorubicin + olaratumab versus single-agent doxorubicin (26.5 vs 14.7 months; hazard ratio [HR]: 0.46; 95% CI: 0.30–0.71; p = 0.0003) [40]. This combination has now received a positive opinion for marketing authorization in the European countries and accelerated approval in the USA [44]. Likewise, a Phase II randomized trial PICASSO of palifosfamide + doxorubicin versus doxorubicin alone in first and second line therapy of STS displayed significantly improved median PFS for combination versus doxorubicin alone (7.8 vs 4.4 months) in 62 evaluable patients (HR: 0.43; 95% CI: 0.19–0.95; p = 0.019) [45]. These and other new drugs or combinations are currently evaluated in clinical trials [46] and may be one day, they will become first-line treatments of advanced or metastatic LMS.

## Conclusion

The presented case demonstrates failure of doxorubicin and combination of gemcitabine with docetaxel to induce response or stabilize disease in LMS of the stomach metastasizing to the liver. Treatment with pazopanib resulted in stable disease within 10 weeks after the initiation of the therapy.

## Future perspective

Leiomyosarcomas have become increasingly recognized as a heterogeneous group of diseases with diverse molecular profiles and variable drug responses and disease outcomes. However, current chemotherapy regimens do not reflect this reality, and their selection for individual patients is not informed by molecular profiles of their tumors. Nevertheless, we expect major improvements in the treatment of LMS patients in the near future, which will be made possible owing to expanding group of prospective drugs presently evaluated for LMS, combined with better insight into their mode of action, and better understanding of disease-relevant gene expression and DNA sequence variants, which are shared across or unique for individual LMS cases. We also speculate that application of omics methods to leiomyosarcoma research will enhance our understanding of its etiology.

**Executive summary**

- Leiomyosarcoma of the stomach is a very rare malignancy. Most previously reported cases actually represent gastrointestinal stromal tumors of the stomach, which display considerably different biology and clinical behavior.
- Leiomyosarcoma of the stomach diagnosed in 47-year-old man metastasized to the liver 2 years after his disease-free status following initial surgery + adjuvant chemotherapy. Further progression was not impeded by monotherapy with doxorubicin and combination therapy with docetaxel–gemcitabine, but targeted therapy with pazopanib resulted in stable disease.
- Response to existing chemotherapy is rather limited in leiomyosarcoma. Several new cytotoxic and targeted therapies are currently being evaluated for the treatment of leiomyosarcoma; however, future progress will likely have to reflect disease heterogeneity and its various molecular profiles.
